# Bidirectional Interplay Between Periodontitis and Alzheimer’s Disease: Mechanisms, Clinical Management, and Preventive Strategies

**DOI:** 10.7759/cureus.117264

**Published:** 2026-09-30

**Authors:** Naif A Alwithanani

**Affiliations:** 1 Oral and Maxillofacial Surgery and Diagnostic Sciences Department, Taif University, Taif, SAU

**Keywords:** alzheimer disease, cognitive dysfunction, neuroinflammation, oral microbiota, periodontitis

## Abstract

Alzheimer’s disease (AD) is a major neurodegenerative disorder, whereas periodontitis is a highly prevalent chronic inflammatory disease that may have important systemic consequences. Increasing evidence suggests a bidirectional relationship between periodontitis and AD, with periodontal inflammation potentially contributing to cognitive decline and neurodegeneration, while cognitive and motor impairment may simultaneously worsen oral hygiene and periodontal health. This narrative review synthesizes current evidence regarding the epidemiological association, potential biological mechanisms, clinical implications, and emerging preventive and therapeutic strategies linking the two conditions. Evidence indicates that periodontitis is associated with an increased risk of cognitive impairment and AD, although causality remains unproven. Proposed mechanisms include translocation of periodontal pathogens, particularly *Porphyromonas gingivalis*, systemic inflammation and neuroinflammation, blood-brain barrier disruption, oxidative stress, microbiome alterations, and effects on amyloid-β and tau pathology. In patients with AD, progressive cognitive impairment can compromise oral self-care and increase dependence on caregivers, contributing to periodontal deterioration. Early periodontal assessment, individualized treatment, caregiver-supported oral hygiene, regular maintenance, and interdisciplinary care may therefore be clinically beneficial. Emerging approaches targeting periodontal pathogens, gingipains, and the oral microbiome are promising but require stronger clinical validation. Overall, maintaining periodontal health represents a potentially modifiable component of comprehensive strategies for reducing the oral and systemic burden associated with AD.

## Introduction and background

Alzheimer’s disease (AD) is the leading cause of dementia, accounting for 60-70% of cases and affecting an estimated 57 million people worldwide. With the aging population, this number is projected to rise substantially in the coming decades, imposing profound clinical, social, and economic burdens [1]. Periodontitis, a chronic inflammatory disease of tooth-supporting tissues driven by dysbiotic oral microbiota, remains highly prevalent. Severe periodontitis alone affects more than one billion people globally, with an age-standardized prevalence of approximately 12.5% [2].

Growing epidemiological evidence indicates a consistent association between periodontitis and increased risk of cognitive decline, mild cognitive impairment, and AD, with risk estimates typically ranging from 1.5- to 2-fold or higher after long-term exposure [3,4]. Importantly, this relationship appears bidirectional. Periodontitis may contribute to AD pathogenesis through systemic inflammation, translocation of periodontal pathogens (notably* Porphyromonas gingivalis *and its gingipains), disruption of the blood-brain barrier, neuroinflammation, and promotion of amyloid-β and tau pathology [3,5]. Conversely, progressive cognitive impairment and loss of fine motor skills in patients with AD impair oral hygiene and self-care, thereby exacerbating periodontal inflammation and creating a self-reinforcing cycle [6].

This bidirectional interplay positions the oral-brain axis as a critical interface in aging-related neurodegeneration [3,6]. Periodontitis is increasingly viewed as a potentially modifiable risk factor, raising the possibility that periodontal therapy and improved oral health management could influence AD trajectories or improve the quality of life in affected individuals [7]. This narrative review synthesizes current evidence on the bidirectional relationship between periodontitis and AD. It examines epidemiological associations, mechanistic pathways linking oral and brain pathology, implications for clinical management of periodontal disease in patients with cognitive impairment, and emerging preventive and therapeutic strategies. By integrating these perspectives, the review aims to inform interdisciplinary approaches that address both oral and neurological health in the aging population.

## Review

Methodology

A narrative approach was deliberately chosen over a systematic review or meta-analysis for several reasons. First, the topic spans multiple domains such as epidemiology, molecular pathogenesis, oral-brain axis biology, clinical dentistry, and geriatric neurology, which are heterogeneous in study design (observational cohorts, case-control studies, animal models, in vitro experiments, and interventional trials) [3,8]. Second, the evidence base is rapidly evolving and includes both associative and mechanistic data that cannot be meaningfully pooled quantitatively without substantial risk of bias and heterogeneity. Third, a narrative format permits integration of biological plausibility, clinical context, and emerging therapeutic concepts in a coherent, reader-friendly manner, which is particularly valuable for an interdisciplinary audience of periodontists, neurologists, and geriatricians.

A comprehensive literature search was performed in PubMed/MEDLINE, Scopus, Web of Science, and Google Scholar for articles published between January 2000 and July 2026. Search terms combined Medical Subject Headings (MeSH) and free-text keywords: “periodontitis” OR “periodontal disease” OR “Porphyromonas gingivalis” AND “Alzheimer’s disease” OR “dementia” OR “cognitive decline” OR “neuroinflammation” OR “oral-brain axis,” together with related terms such as “gingipains,” “systemic inflammation,” “bidirectional,” and “periodontal therapy.” Additional relevant papers were identified by screening the reference lists of key reviews and recent primary studies.

The inclusion criteria encompassed original research (human observational studies, clinical trials, animal models), mechanistic investigations, and high-quality narrative, scoping, or systematic reviews published in English that addressed the association, mechanisms, or clinical implications of periodontitis and AD. The exclusion criteria included case reports, conference abstracts without full text, non-English publications, and studies focused exclusively on other neurodegenerative diseases without specific AD data. Titles and abstracts were screened for relevance, followed by full-text assessment. Priority was given to recent high-impact reviews and primary studies that provided epidemiological risk estimates, pathogen detection in brain tissue, experimental evidence of neuroinflammation or amyloid/tau pathology, or data on periodontal interventions.

The selected literature was synthesized thematically under four major headings: epidemiological evidence of association and bidirectionality, proposed biological mechanisms, clinical management of periodontitis in patients with AD, and preventive or therapeutic strategies. No formal quality scoring or risk-of-bias tool was applied, consistent with the narrative design; instead, the strengths and limitations of the evidence were discussed qualitatively throughout the review.

Epidemiological evidence of the bidirectional association

A substantial body of observational research supports a positive association between periodontitis and the risk of cognitive impairment, dementia, and AD [3,6-8]. Multiple cohort, case-control, and cross-sectional studies have reported elevated risks, with odds ratios (ORs) typically ranging from approximately 1.67 to 2.17 after adjustment for common confounders, such as age, sex, education, smoking, and cardiovascular comorbidities [9].

A retrospective, population-based, matched-cohort study reported that individuals with chronic periodontitis had a 1.707-fold higher risk of subsequently developing AD compared with matched controls without chronic periodontitis (adjusted HR 1.707, 95% CI 1.152-2.528; p = 0.0077) [10]. Similar findings have been replicated in other large databases, indicating that both the presence and duration or severity of periodontal disease contribute to elevated risk. An umbrella review synthesizing multiple systematic reviews confirmed consistent associations, with ORs ranging from 1.67 to 2.17 and risk ratios from 1.11 to 2.26 across included studies [9].

Recent meta-analyses and scoping reviews have reinforced these observations. Pooled estimates from observational studies involving hundreds of thousands of participants have yielded overall ORs of approximately 2.67 (95% CI 1.41-5.06), although substantial heterogeneity remains, reflecting differences in diagnostic criteria for both periodontitis and AD, follow-up duration, and residual confounding [11]. Longitudinal data further suggest that pre-existing periodontitis is linked to accelerated cognitive decline once AD is diagnosed, with some reports indicating up to a six-fold faster rate of deterioration [12].

Importantly, the relationship appears bidirectional. Patients with established AD exhibit a markedly higher prevalence of periodontitis than cognitively healthy controls [13]. Cognitive decline impairs oral hygiene practices, reduces the frequency of dental visits, and diminishes the fine-motor skills required for effective plaque control, thereby promoting periodontal inflammation and progression [14]. One retrospective cohort study reported that individuals with AD had a 1.7-fold higher risk of developing periodontitis compared with matched controls without AD [10]. Clinical observations also indicate that the severity of periodontitis tends to worsen from mild cognitive impairment to frank dementia, supporting reciprocal exacerbation [13,15].

While these epidemiological findings are robust in demonstrating an association, they do not yet establish definitive causality. Most evidence remains observational, and residual confounding by shared risk factors, such as socioeconomic status, systemic inflammation, diabetes, or lifestyle, cannot be fully excluded. Nevertheless, the consistency across diverse populations, the presence of dose-response patterns, and the emerging bidirectional data collectively strengthen the rationale for regarding periodontitis as a potentially modifiable contributor to AD risk and progression.

Proposed biological mechanisms

Two principal, non-mutually exclusive frameworks have been proposed to explain how periodontitis may contribute to AD pathogenesis: the microbial involvement and inflammatory cascade theories [7,9]. In the microbial involvement pathway, keystone periodontal pathogens, particularly* Porphyromonas (P.) gingivalis*, and their virulence factors can access the central nervous system. *P. gingivalis* DNA and its cysteine proteases (gingipains) have been detected in post-mortem brain tissue and cerebrospinal fluid of patients with AD, often colocalized with amyloid-β plaques, tau tangles, and neurons [16,17]. Gingipains can degrade tight-junction proteins, compromise blood-brain barrier integrity, promote amyloid-β production and aggregation, and induce tau hyperphosphorylation and truncation. Experimental oral infection with *P. gingivalis* in wild-type mice recapitulates several AD-like features, including neuroinflammation, amyloid-β accumulation, tau pathology, and neuronal loss [17,18]. Lipopolysaccharide (LPS) from periodontal bacteria further activates microglial Toll-like receptors, amplifying local inflammatory responses [19].

The inflammatory cascade theory emphasizes the generation of chronic low-grade systemic inflammation due to periodontitis. Elevated circulating levels of pro-inflammatory cytokines (IL-1β, IL-6, and TNF-α) and acute-phase proteins can cross or disrupt the blood-brain barrier, activate microglia, and sustain neuroinflammation [20]. This peripheral-to-central inflammatory signaling is thought to exacerbate amyloid and tau pathology and accelerate synaptic dysfunction [21]. Ahmed et al. synthesized the current evidence on the oral-brain axis linking periodontitis and AD [21]. They described two core pathways: an inflammatory cascade in which periodontal cytokines (IL-1β and TNF-α) disrupt the blood-brain barrier and promote amyloid-β and tau pathology, and a microbial pathway in which *P. gingivalis *reaches the brain via the bloodstream or trigeminal nerve and activates microglia. The authors also emphasized the bidirectional nature of the relationship, noting that cognitive decline worsens oral hygiene and sustains periodontal disease.

Papadakis et al. reviewed the role of oxidative stress as a key mechanistic link between periodontitis and AD [22]. They summarized animal and human evidence showing that periodontal inflammation increases reactive oxygen species, elevates lipid peroxidation markers, such as malondialdehyde, and oxidative DNA damage markers including 8-hydroxy-2′-deoxyguanosine, while depleting important antioxidant enzymes such as superoxide dismutase and glutathione peroxidase. These changes promote neuroinflammation, mitochondrial dysfunction, and cognitive decline. Human data further indicate lower total antioxidant capacity and higher systemic oxidative stress markers, such as advanced oxidation protein products, in patients with both conditions. The authors suggest that improving periodontal health may help reduce oxidative damage and lower the risk of cognitive decline.

Emerging evidence implicates the oral-gut-brain axis. Periodontal pathogens and inflammatory mediators can alter gut microbiota composition, increase intestinal permeability, and thereby amplify systemic and central inflammatory responses [23]. Conversely, AD-related neurodegeneration exerts reciprocal effects on the periodontium. Amyloid-β can activate osteoclasts via RAGE/NF-κB signaling and suppress osteoblastogenesis; autonomic dysregulation (sympathetic hyperactivity) promotes RANKL-mediated bone resorption; and endocrine changes (hypercortisolism, sex-hormone deficiency, and insulin resistance) further disturb periodontal bone homeostasis [13]. Cognitive and motor decline secondary to AD simultaneously impairs oral hygiene, creating a self-reinforcing cycle of periodontal deterioration and systemic inflammation. Collectively, these interlocking mechanisms-microbial translocation, systemic and neuroinflammation, barrier disruption, and bidirectional bone-brain interactions-provide biological plausibility for the observed epidemiological associations and highlight multiple potential targets for intervention.

Clinical management of periodontitis in patients with AD

Managing periodontitis in individuals with AD presents unique clinical challenges because of progressive cognitive decline, reduced motor coordination, behavioral changes, and limited ability to maintain daily oral hygiene [24]. As the disease advances from mild cognitive impairment to moderate and severe dementia, patients become increasingly dependent on caregivers for oral care; however, plaque accumulation, gingival inflammation, and periodontal destruction often worsen [25]. A comprehensive oral assessment should be performed as early as possible after the diagnosis of cognitive impairment. This allows the establishment of a baseline periodontal status and facilitates proactive intervention while the patient can still cooperate. Treatment planning must be individualized, taking into account the patient’s cognitive stage, cooperation level, medical comorbidities, and the availability of caregiver support [25].

The 2020 U.S. Preventive Services Task Force evidence review examined screening for cognitive impairment in adults aged ≥ 65 years. It found that several brief instruments can detect dementia with reasonable accuracy, but there is no clear evidence that routine screening improves patient or caregiver outcomes or decision-making. Pharmacologic treatments (acetylcholinesterase inhibitors and memantine) produce only small, short-term cognitive benefits of uncertain clinical importance and are associated with more adverse effects. Non-pharmacologic approaches and caregiver-support interventions show modest benefits on caregiver burden and depression. Overall, the review concluded that the benefits of screening and early treatment remain limited and of uncertain clinical significance [26].

Non-surgical periodontal therapy (scaling and root planing) remains the foundation of management and can usually be completed in patients with mild to moderate Alzheimer’s disease, preferably under local anesthesia and with shorter, more frequent appointments to reduce stress and fatigue [27]. Adjunctive antimicrobial agents may be considered in selected cases, although the risk-benefit balance must be carefully weighed in older adults with polypharmacy. Surgical periodontal procedures are generally reserved for patients with good cooperation and early-stage disease.

Maintenance therapy is critical. Recall intervals should be shortened (often every two to three months), and caregivers should receive practical training in daily plaque control techniques, including the use of electric toothbrushes, interdental aids adapted to reduced manual dexterity, and antiseptic rinses when appropriate [25,26]. Behavioral strategies, such as timed oral care routines, visual cues, and positive reinforcement, can improve compliance. In advanced dementia, the focus shifts from definitive periodontal therapy to palliative oral care aimed at controlling infection, preventing pain, reducing aspiration risk, and preserving dignity [26]. Collaboration between dentists, neurologists, geriatricians, and caregivers is essential to integrate oral health into the overall care plan. Emerging observational evidence suggests that successful periodontal treatment may reduce the systemic inflammatory burden and has been associated with favorable effects on markers of brain atrophy and slower cognitive decline in some studies [28]. While definitive randomized interventional data remain limited, maintaining periodontal health is a low-risk and potentially beneficial component of comprehensive Alzheimer’s disease management.

Preventive and therapeutic strategies

Promising interventions aimed at disrupting the oral-brain axis include gingipain inhibitors (such as COR388), nitrate-reducing probiotics, and integrated dental-neurology care pathways. Early observational and preclinical data suggest that targeted reduction of *P. gingivalis* virulence factors and modulation of the oral microbiome may attenuate systemic and neuroinflammatory signalling [19,29]. Oral microbiome screening should be performed in high-risk populations, including apolipoprotein E ε4 carriers and individuals with diabetes, to identify those who may benefit most from intensive periodontal management [30]. Interdisciplinary collaboration between dental professionals, neurologists, and geriatricians is essential to embed oral health strategies within broader AD prevention frameworks. Although large-scale randomized trials are still needed to confirm clinical benefit, these approaches represent low-risk, potentially modifiable targets that warrant prioritization in future research and clinical practice.

Limitations and future directions

Despite growing evidence supporting a bidirectional relationship between periodontitis and AD, several limitations remain. Most human data are observational, making residual confounding and reverse causality difficult to exclude. Diagnostic criteria for both periodontitis and cognitive impairment vary widely across studies, contributing to heterogeneity in risk estimates. Mechanistic insights are largely derived from animal models and post-mortem human tissue, and direct causal evidence in living patients remains limited. Interventional trials examining the effect of periodontal therapy or targeted microbiome modulation on cognitive outcomes remain few and underpowered.

Future research should prioritize large, well-designed prospective cohort studies with standardized periodontal and cognitive assessments, as well as randomized controlled trials evaluating the impact of intensive periodontal treatment, gingipain inhibitors, and oral microbiome interventions on biomarkers of neurodegeneration and clinical cognitive trajectories. Longitudinal studies that incorporate oral microbiome profiling in high-risk groups (apolipoprotein E ε4 carriers and individuals with diabetes) will help identify those most likely to benefit from early preventive strategies. Interdisciplinary collaboration between dental, neurological, and geriatric research communities is essential for advancing this field.

## Conclusions

Current evidence indicates a consistent epidemiological association and a biologically plausible bidirectional interplay between periodontitis and Alzheimer’s disease, mediated by microbial translocation, systemic and neuroinflammation, oxidative stress, and reciprocal effects of neurodegeneration on oral health. Although causality has not been definitively established, periodontitis represents a potentially modifiable factor that can be addressed through routine dental care. Early periodontal assessment, individualized treatment adapted to cognitive stage, caregiver training, and integration of oral health into dementia care pathways are clinically justified. Emerging targeted approaches, including gingipain inhibitors and microbiome modulation, hold promise but require further validation. Maintaining periodontal health should be regarded as a practical and low-risk component of comprehensive strategies aimed at reducing the burden of Alzheimer’s disease in ageing populations.

## References

[1] (2025). 2025 Alzheimer's disease facts and figures. Alzheimers Dement.

[2] Nascimento GG, Alves-Costa S, Romandini M (2024). Burden of severe periodontitis and edentulism in 2021, with projections up to 2050: the Global Burden of Disease 2021 study. J Periodontal Res.

[3] Bouziane A, Lattaf S, Abdallaoui Maan L (2023). Effect of periodontal disease on Alzheimer’s disease: a systematic review. Cureus.

[4] Goyal L, Gupta S, Perambudhuru Y (2023). Association between periodontitis and cognitive impairment in adults. Evid Based Dent.

[5] Leira Y, Domínguez C, Seoane J (2017). Is periodontal disease associated with Alzheimer’s disease? A systematic review with meta-analysis. Neuroepidemiology.

[6] Dziedzic A (2022). Is periodontitis associated with age-related cognitive impairment? The systematic review, confounders assessment and meta-analysis of clinical studies. Int J Mol Sci.

[7] Hu X, Zhang J, Qiu Y, Liu Z (2021). Periodontal disease and the risk of Alzheimer's disease and mild cognitive impairment: a systematic review and meta-analysis. Psychogeriatrics.

[8] Nascimento GG, Leite FR, Mesquita CM, Vidigal MT, Borges GH, Paranhos LR (2023). Confounding in observational studies evaluating the association between Alzheimer's disease and periodontal disease: a systematic review. Heliyon.

[9] Arbildo-Vega HI, Cruzado-Oliva FH, Coronel-Zubiate FT (2025). Association between periodontal disease and Alzheimer's disease: umbrella review. Front Dent Med.

[10] Chen CK, Wu YT, Chang YC (2017). Association between chronic periodontitis and the risk of Alzheimer's disease: a retrospective, population-based, matched-cohort study. Alzheimers Res Ther.

[11] Ridho FM, Irmawati A, Wicaksana AP, Alfatah R, Labib R (2026). Alzheimer's disease and periodontal disease: uncovering the association through systematic review and meta-analysis. Rev Clin Esp (Barc).

[12] Kwon HJ, Ahn JH, Won MH, Kim DW (2026). Chronic periodontitis and systemic inflammation in the elderly: implications for neurodegeneration. J Neuroinflammation.

[13] Zhang W, Li S, Cui Y, Lun Y, Liu H, Li M (2026). The interface of oral and brain health: current insights into the bidirectional relationship between Alzheimer’s disease and periodontitis. CNS Neurosci Ther.

[14] Plachokova AS, Gjaltema J, Hagens ER (2024). Periodontitis: a plausible modifiable risk factor for neurodegenerative diseases? A comprehensive review. Int J Mol Sci.

[15] Watts A, Crimmins EM, Gatz M (2008). Inflammation as a potential mediator for the association between periodontal disease and Alzheimer's disease. Neuropsychiatr Dis Treat.

[16] Shawkatova I, Durmanova V, Javor J (2025). Alzheimer’s disease and Porphyromonas gingivalis: exploring the links. Life (Basel).

[17] Dominy SS, Lynch C, Ermini F (2019). Porphyromonas gingivalis in Alzheimer's disease brains: evidence for disease causation and treatment with small-molecule inhibitors. Sci Adv.

[18] Haditsch U, Roth T, Rodriguez L (2020). Alzheimer’s disease-like neurodegeneration in Porphyromonas gingivalis infected neurons with persistent expression of active gingipains. J Alzheimers Dis.

[19] Rozenblum G, Ait-Aissa K, Zahran G (2025). Unraveling the oral microbiome's role in Alzheimer's disease: from pathophysiology to therapeutic potential. Alzheimers Dement.

[20] Kong L, Li J, Gao L (2025). Periodontitis-induced neuroinflammation triggers IFITM3-Aβ axis to cause Alzheimer's disease-like pathology and cognitive decline. Alzheimers Res Ther.

[21] Ahmed MA, Ahmed AH, Elmoghazy NH, Shaheraldin AA, Ghalwash D (2025). Pathophysiological insights into the oral-brain axis: evidence-based mechanisms connecting periodontitis and Alzheimer’s disease. Eur J Neurosci.

[22] Papadakis KA, Doufexi AE, Kalamaki MS, Bourazanas E, Lymperaki E (2025). The role of oxidative stress in the relationship between periodontitis and Alzheimer’s disease: a review of the literature. J Pers Med.

[23] Zhang P, Liu Y, Jin X (2025). Alzheimer's disease-like pathology induced by Porphyromonas gingivalis in middle-aged mice is mediated by NLRP3 inflammasome via the microbiota-gut-brain axis. J Alzheimers Dis.

[24] Ryder MI, Xenoudi P (2021). Alzheimer disease and the periodontal patient: new insights, connections, and therapies. Periodontol 2000.

[25] Silva MG, Vasconcelos MB, Silva RG, Feitosa MÁL (2026). Oral health in Alzheimer’s disease: a life-course perspective on clinical management and caregiver support. Spec Care Dentist.

[26] Patnode CD, Perdue LA, Rossom RC, Rushkin MC, Redmond N, Thomas RG, Lin JS (2020). Screening for cognitive impairment in older adults. Updated evidence report and systematic review for the US Preventive Services Task Force. JAMA.

[27] Gharaibeh S, Alsabbah A, Alloubani A, Gharaibeh A (2025). Reciprocal interactions between periodontal disease and Alzheimer’s disease: implications for mutual triggering, exacerbation, and treatment interventions-a comprehensive review of the literature. Neurol Int.

[28] Inchingolo AM, Inchingolo AD, Piras F (2025). Oral health and cognitive decline: a systematic review of the periodontitis-Alzheimer’s connection. Appl Sci (Basel).

[29] Pedrosa ME, Martin V, Fernandes MH, Gomes PS (2025). Gingipain inhibitors as an innovative therapy for periodontal and associated-systemic diseases: a systematic review. Clin Oral Investig.

[30] Li F, Liang X, Li J (2026). Oral microbiome perturbations link periodontal health to cognitive ageing in a large community cohort. EBioMedicine.

